# Comments on Rajizadeh et al. (2023) ‘Comparison of preventive and therapeutic effects of continuous exercise on acute lung injury induced with methotrexate’

**DOI:** 10.1113/EP092385

**Published:** 2025-01-20

**Authors:** Mehran Hosseini

**Affiliations:** ^1^ Department of Anatomical Sciences Birjand University of Medical Sciences Birjand Iran

1

In the paper by Rajizadeh et al. (2023), the authors investigated the effects of continuous training (CT) exercise on acute lung injury (ALI) induced by methotrexate (Mtx) in rats. Given the known side effects of Mtx, including oxidative stress and lung inflammation, this research aimed to explore the potential protective role of exercise. The results indicated that Mtx administration significantly increased oxidative stress and inflammatory markers, such as tumor necrosis factor‐alpha (TNF‐α) and malondialdehyde (MDA), while decreasing antioxidant levels. CT, particularly when administered as a pretreatment, effectively mitigated these adverse effects, achieving notable reductions in TNF‐α, MDA and myeloperoxidase levels, in addition to increases in interleukin‐10 and other protective factors. The authors claimed that histological evaluations confirmed that CT improved lung tissue pathology following injury.

However, the study by Rajizadeh et al. (2023) contains fundamental flaws that raise significant concerns regarding the accuracy of the results. The most critical issue is the use of adipose tissue in the immunohistochemical (IHC) analyses instead of lung tissue. The IHC micrographs presented in their figure 7 for the control and CT groups were derived from adipose tissue, whereas other micrographs were from lung tissue. This discrepancy undermines the reliability of the IHC findings and calls into question the accuracy of other tissue‐based results, because using adipose tissue in ELISA, antioxidant and molecular studies might similarly compromise the validity of those measurements.

Adipose tissue functions primarily in energy storage and metabolic regulation, characterized by prominent lipid droplets and a relatively simple cellular architecture. In contrast, lung tissue is specialized for respiratory function, featuring a complex arrangement of cells and structures that facilitate efficient gas exchange. Key components of lung tissue include alveoli, bronchioles and blood vessels. The alveolar walls are notably thin and consist of two types of pneumocytes: type I pneumocytes are essential for gas exchange, while type II pneumocytes produce surfactant to reduce surface tension (Mescher, 2018). The differences in cellular composition, structural organization and functional specialization underscore the distinct roles these tissues play within the body.

Additionally, there are inconsistencies in the method subsections related to histology and IHC, suggesting that some content might have been copied from a study focused on immunocytochemistry. In the IHC method (subsection 2.16), cell evaluation is mentioned multiple times, despite the study focusing on tissue expression of Caspase 3. The IHC images imply the use of DAB (3,3′‐diaminobenzidine) for visualization, as evidenced by the developed brown colour; however, the Methods section (2.16) refers to Hoechst and immunofluorescent dyes (‘The samples were washed three times with PBS and stained at room temperature with H‐Hoc (5 g/mL) for 15 min. The slides were imaged by reverse fluorescence microscopy.’), which are not observed in the IHC micrographs. In subsections 2.6 and 2.15, it is stated that the right lung (given that the left lung was used for collection of bronchoalveolar lavage fluid) was used for histological and IHC evaluations. However, subsection 2.16 mentions that the left lung was used for IHC. It is well known that tissue preparation processes leading up to slide preparation are essentially the same for histological and IHC evaluations; the differences arise only during the staining or incubation stages with antibodies. The inconsistencies in subsections 2.15 and 2.16 suggest that the authors might not have been aware of this.

Further concerns include discrepancies in the reported lethal doses of ketamine and xylazine across various subsections of the Methods (subsections 2.6, 2.7 and 2.9), in addition an incomplete last sentence of subsection 3.6.

Last but not least, in the study by Rajizadeh et al. (2023), five experimental groups were allocated. Among them, three groups (ALI, Pre‐CT and Post‐CT) received a single Mtx injection (20 mg/kg) to induce lung damage. The sampling time for the ALI and Pre‐CT groups was 5 days following Mtx administration, and the sampling time for the Post‐CT group was 68 days after Mtx administration. Surprisingly, data from all groups were compared with each other. It would have been more appropriate to include another ALI model group, in which the interval between Mtx administration and sampling was also 68 days.

Despite the significant advancements in molecular biology and the development of sophisticated techniques, histological evaluations remain a cornerstone of both basic research and diagnostic practice. However, the reliability of these evaluations can be severely compromised if conducted by individuals lacking the requisite expertise, potentially leading to errors. This commentary emphasizes the importance of involving qualified pathologists or histologists in studies using histological assessments, because their specialized knowledge is vital for ensuring the accuracy and validity of findings.

## AUTHOR CONTRIBUTIONS

Sole author.

## CONFLICT OF INTEREST

There are neither ethical nor financial conflicts of interest involved in the manuscript.

## FUNDING INFORMATION

None.
